# Supplementary figures and images for: Litter expansion alters metabolic homeostasis in a sex specific manner
Source: PLoS One. 2021 Sep 29;16(9):e0237199. doi: 10.1371/journal.pone.0237199 (PMC8480909; doi:10.1371/journal.pone.0237199)

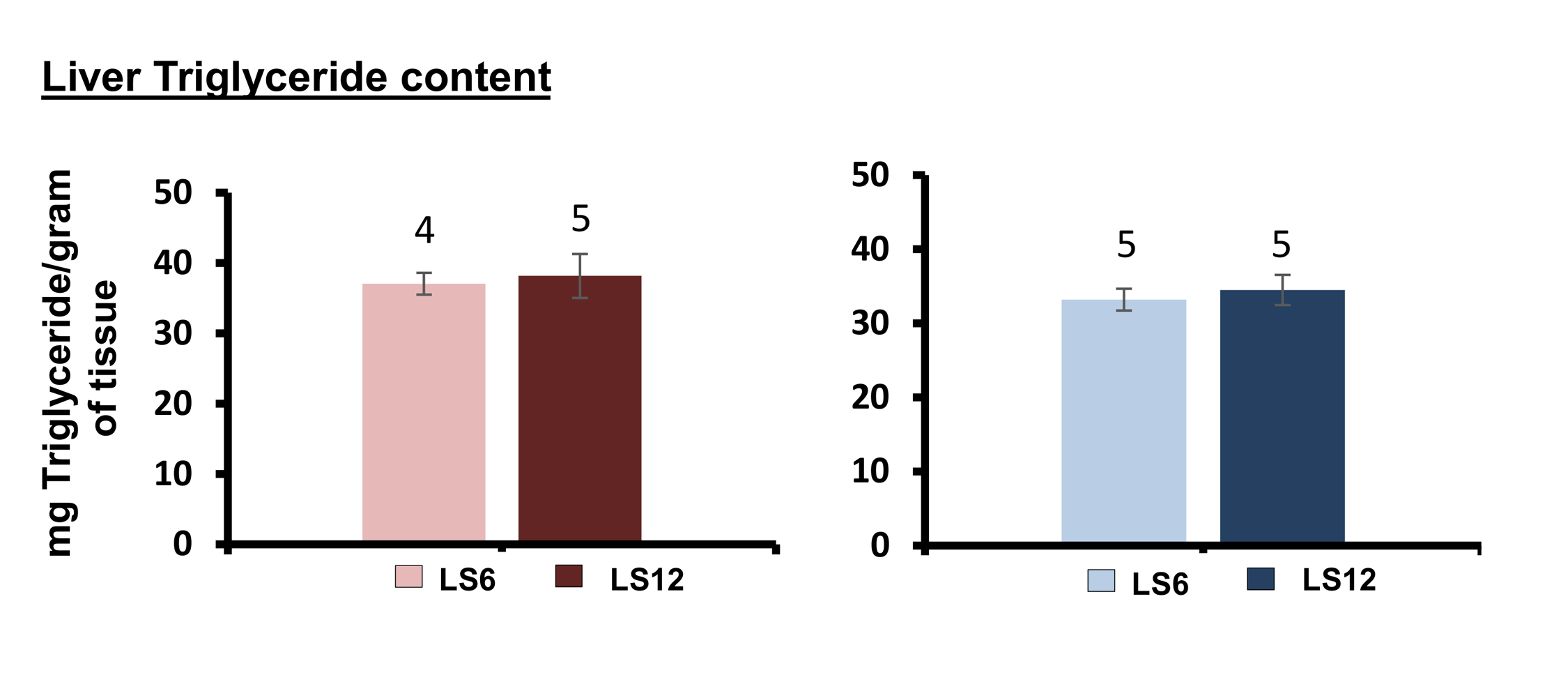

Supplement: S1 Fig — The triglyceride content of the liver obtained from LS6 and LS12 were measured in female and male mice at 15 months of age. The liver triglyceride content data represented are the mean ± SEM from 4–5 mice per group. All groups were compared to control LS6 and were statistically analyzed by one-way ANOVA with Tukey’s and FDR with Benjamini & Hochberg multiple correction test (*p<0.05). (TIF) [file pone.0237199.s001.tif]

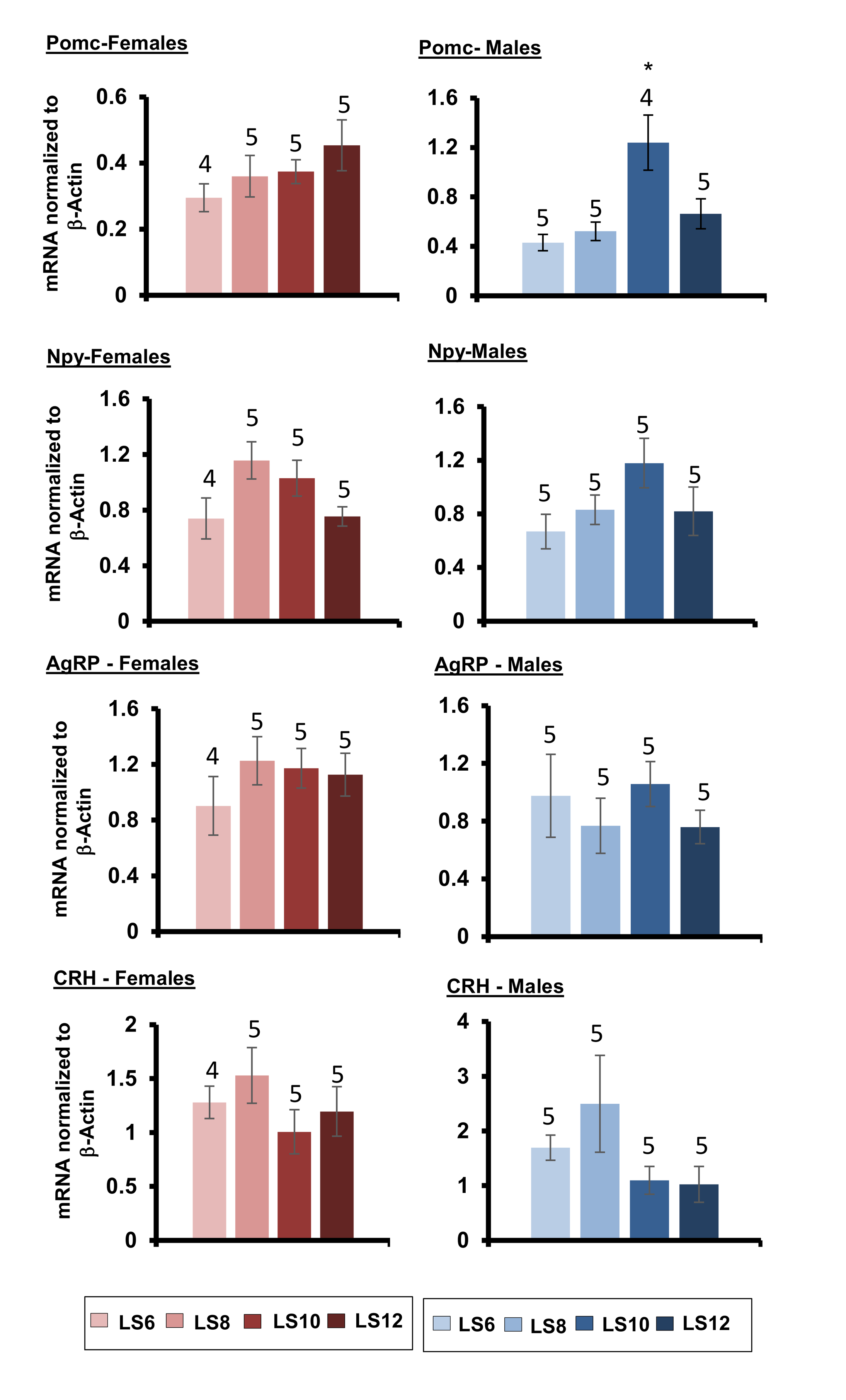

Supplement: S2 Fig — Levels of mRNA of Pomc, Npy, AgRP and CRH genes were measured in the hypothalamus of female and male mice from various litter sizes (LS6, LS8, LS10 and LS12 pups/litter) at 15 months of age. Data represented are the mean ± SEM from 4–5 mice per group. All groups were compared to control LS6 and were statistically analyzed by one-way ANOVA with Tukey’s and FDR with Benjamini & Hochberg multiple correction test (*p<0.05). (TIF) [file pone.0237199.s002.tif]

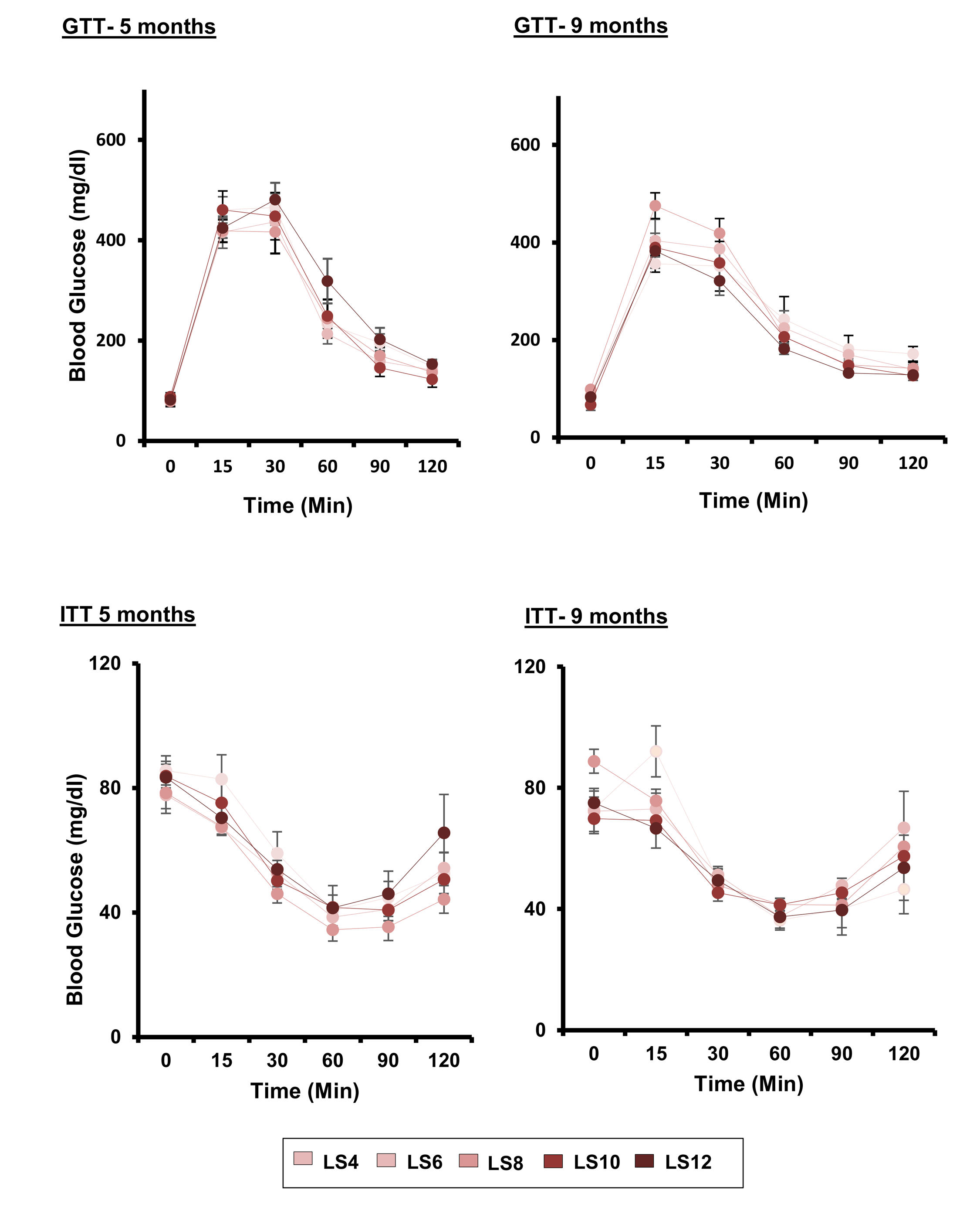

Supplement: S3 Fig — Glucose tolerance and insulin tolerance was determined after an overnight fast of mice at 5 and 9 months of age. Data represented are the mean ± SEM from 4–5 mice per group. All groups were compared to control LS6 and were statistically analyzed by one-way ANOVA with Tukey’s and FDR with Benjamini & Hochberg multiple correction test (*p<0.05). (TIF) [file pone.0237199.s003.tif]

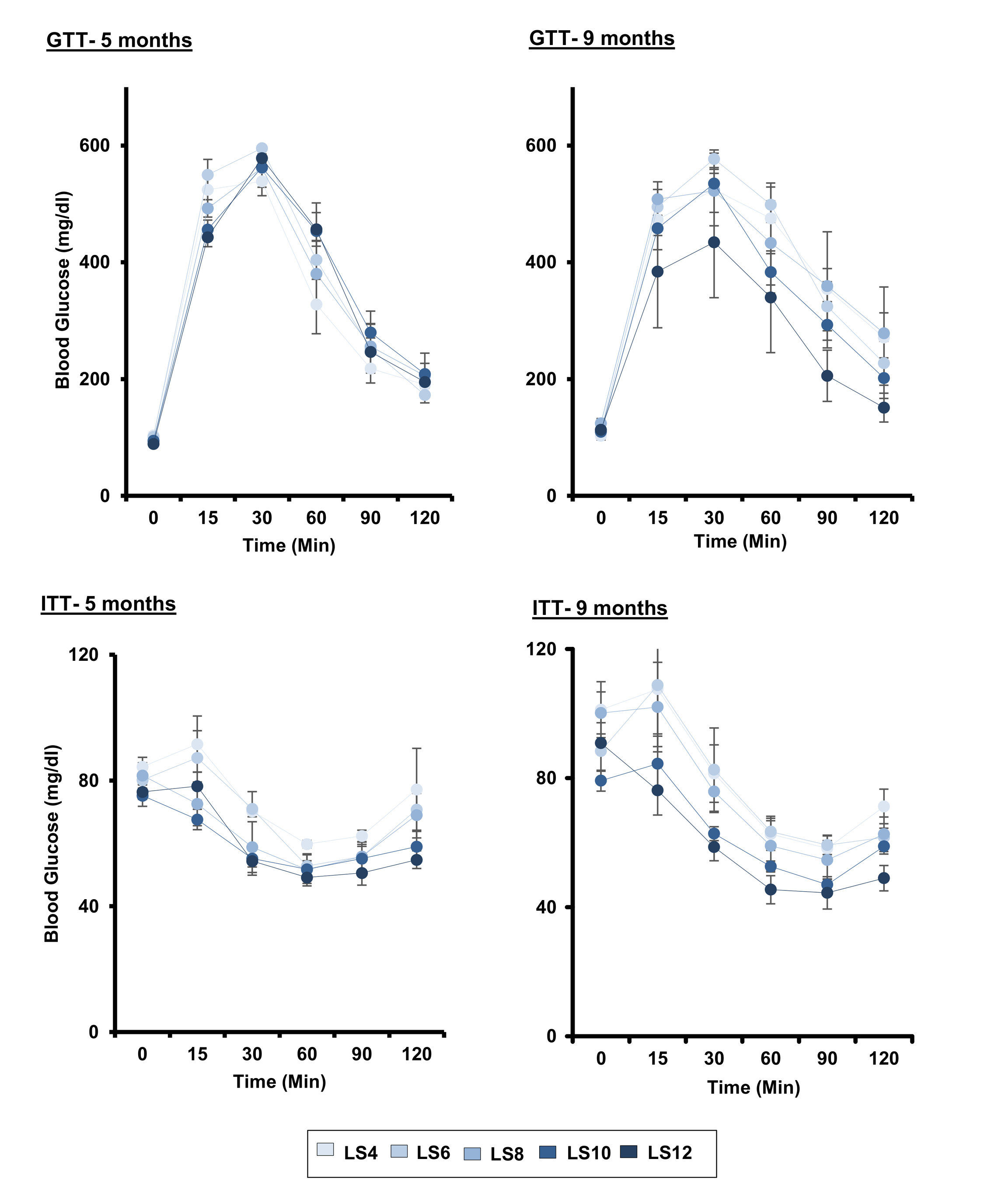

Supplement: S4 Fig — Glucose tolerance and insulin tolerance was determined after an overnight fast of mice at 5 and 9 months of age. Data represented are the mean ± SEM from 4–5 mice per group. All groups were compared to control LS6 and were statistically analyzed by one-way ANOVA with Tukey’s and FDR with Benjamini & Hochberg multiple correction test (*p<0.05). (TIF) [file pone.0237199.s004.tif]

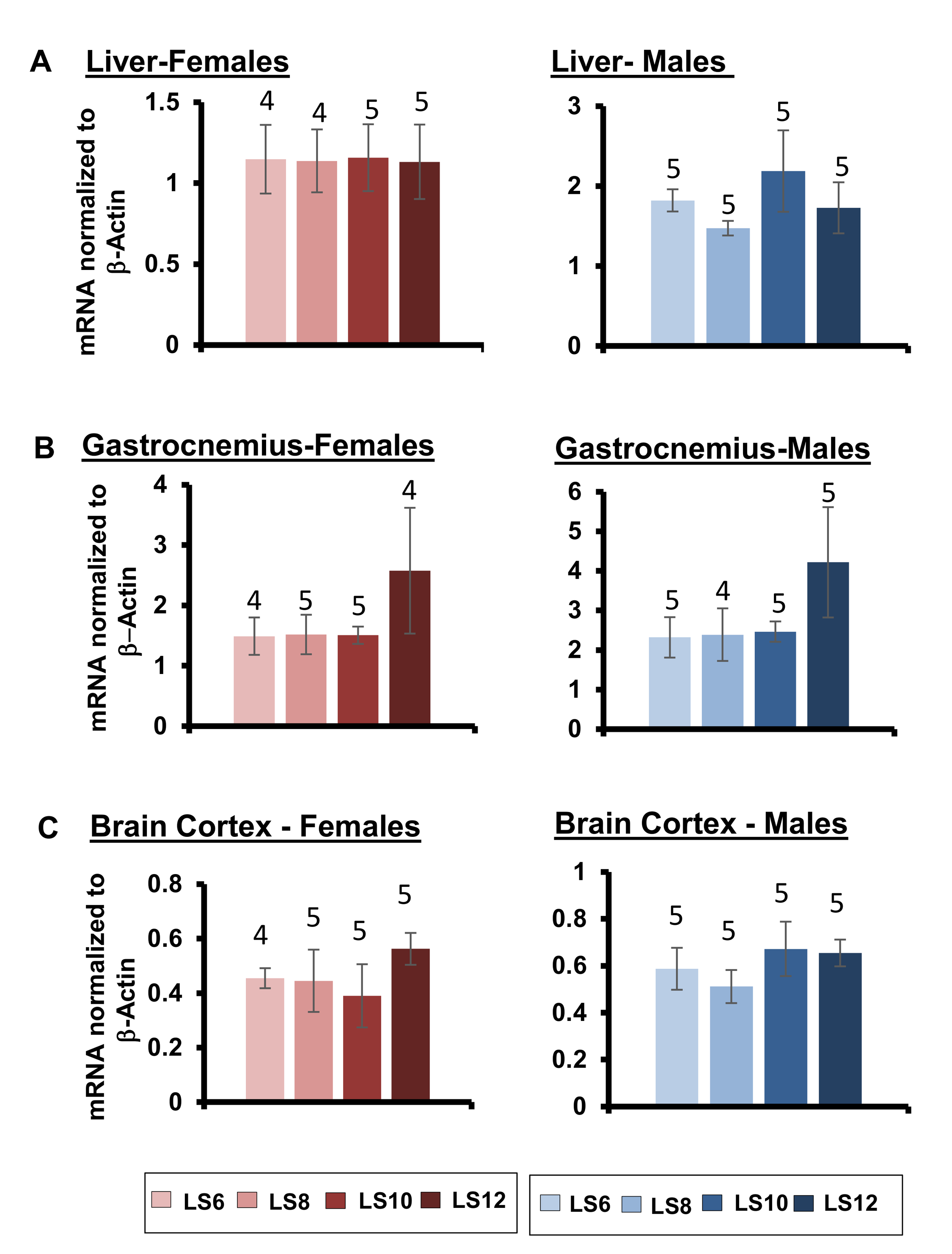

Supplement: S5 Fig — mRNA levels of IGF-1 in the liver (A), gastrocnemius (B) and brain cortex (C) of female and male mice from various litter sizes (LS6, LS8, LS10 and LS12 pups/litter) were measured at 15 months of age. Data represented are the mean ± SEM from 4–5 mice per group. All groups were compared to control LS6 and were statistically analyzed by one-way ANOVA with Tukey’s and FDR with Benjamini & Hochberg multiple correction test (*p<0.05). (TIF) [file pone.0237199.s005.tif]
