# Supplementary material for: Litter expansion alters metabolic homeostasis in a sex specific manner
Source: PLoS One. 2021 Sep 29;16(9):e0237199. doi: 10.1371/journal.pone.0237199 (PMC8480909; doi:10.1371/journal.pone.0237199)
Supplement: S1 Table — *One mouse dead during shipment from Jackson laboratories, **One mouse found dead or euthanized due to dermal injuries during the study, ***Three mice found dead or euthanized due to dermal injuries at various times during the study. (DOCX) [file pone.0237199.s006.docx]

| **S1 Table: Number of Lactating Dams** | | | | |
| --- | --- | --- | --- | --- |
| **LS Group** | **Total number of lactating dams in each group** | | **Number of Males/Litter/dam** | **Number of Females/Litter/dam** |
| LS4 | 4 | Dam 1 | 3 | 1 |
|  |  | Dam 2 | 1 | 3 |
|  |  | Dam 3 | 3** | 1 |
|  |  | Dam 4 | 1* | 3 |
| LS6 | 3 | Dam 1 | 3 | 3 |
|  |  | Dam 2 | 4 | 2 * |
|  |  | Dam 3 | 5 | 1** |
| LS8 | 2 | Dam 1 | 7 * | 1 ** |
|  |  | Dam 2 | 3 | 5 |
| LS10 | 2 | Dam 1 | 6*** | 4 |
|  |  | Dam 2 | 5 | 5 |
| LS12 | 2 | Dam 1 | 6 | 6 |
|  |  | Dam 2 | 6 | 6 |

*One mouse dead during shipment from Jackson laboratories

**One mouse found dead or euthanized due to dermal injuries during the study

***Three mice found dead or euthanized due to dermal injuries at various times during the study
